# Mesenchymal stem cells in combination with erythropoietin repair hyperoxia-induced alveoli dysplasia injury in neonatal mice via inhibition of TGF-β1 signaling

**DOI:** 10.18632/oncotarget.9314

**Published:** 2016-05-12

**Authors:** Yun Luan, Luan Zhang, Sun Chao, Xiaoli Liu, Kaili Li, Yibiao Wang, Zhaohua Zhang

**Affiliations:** ^1^ Central Research Laboratory, The Second Hospital of Shandong University, Jinan, China; ^2^ Department of Pediatrics, The Second Hospital of Shandong University, Jinan, China; ^3^ Department of Hematology, The Second Hospital of Shandong University, Jinan, China

**Keywords:** BPD, MSCs, EPO, TGF-β1, ETM

## Abstract

The aim of the present study is to investigate the protection effects of bone marrow mesenchymal stem cells (MSCs) in combination with EPO against hyperoxia-induced bronchopulmonary dysplasia (BPD) injury in neonatal mice. BPD model was prepared by continuous high oxygen exposure, 1×10^6^ bone marrow MSCs and 5000U/kg recombinant human erythropoietin (EPO) were injected respectively. Results showed that administration of MSCs, EPO especially MSCs+EPO significant attenuated hyperoxia-induced lung damage with a decrease of fibrosis, radical alveolar counts and inhibition of the occurrence of epithelial-mesenchymal transition (EMT). Furthermore, MSCs+EPO co-treatment more significantly suppressed the levels of transforming growth factor-β1(TGF-β1) than MSCs or EPO alone. Collectively, these results suggested that MSCs, EPO in particular MSCs+EPO co-treatment could promote lung repair in hyperoxia-induced alveoli dysplasia injury via inhibition of TGF-β1 signaling pathway to further suppress EMT process and may be a promising therapeutic strategy.

## INTRODUCTION

Bronchopulmonary dysplasia (BPD), a serious and common complication of prematurity chronic respiratory disease in infants that follows ventilator and oxygen therapy for acute respiratory failure [1]. Patients with BPD, suffer from lung fibrosis secondary to myofibroblast-mediated excessive extracellular matrix (ECM) deposition and destruction of lung architecture. It is widely believed that transforming growth factor β1 (TGF-β1) plays a key role in pulmonary fibrosis, can induce fibroblast migration, proliferation and differentiation of myofibroblasts, and deposition of ECM [2]. TGF-β1 is also known to strongly induce epithelial-mesenchymal transition (EMT) that contributes to the generation and accumulation of fibroblasts and myofibroblasts responsible for excessive ECM deposition. This process is accompanied by the loss of intercellular cohesion and epithelial makers [3]. EMT is a biologically important process that allows for tissue remodeling in the developing embryo. TGF-β1 induces EMT of alveolar epithelial cells to myofibroblasts both *in vitro* and *in vivo* [4]. Recent studies have demonstrated that the majority of myofibroblast-like cells in experimental injury are the result of alveolar EMT [5]. TGF-β1 induces EMT via regulation of Smads and non-Smads signaling pathways [6], increased expression of Smad2 induces EMTs, while increased expression of Smad7 blocks TGF-β-induced EMT in multiple tissue. There exists strong evidence that alveolar EMT is primarily mediated by local production and activation of TGF-β1 [7, 8], however, using TGF-β1 inhibitors for the treatment of pulmonary fibrosis is little and the mechanism have not been identified.

So far, no effective therapy is available for BPD, a common and serious complication with high morbidity and mortality. Interest has recently been focused on the potential therapeutic effect of mesenchymal stem cells (MSCs) in the recovery of hyperoxia-induced BPD [9]. Although many efforts have been made to investigate MSCs in the treatment of BPD, the mechanism is not yet clear. Recently, erythropoietin (EPO) was studied in the treatment of BPD for their novel pharmacological effect [10]. Treatment with EPO during hyperoxia exposure is associated with improved alveolar structure, enhanced vascularity, and decreased fibrosis. In the present study, we explored the different effects of MSCs, EPO alone or MSCs+EPO in the treatment of BPD. Two weeks after operation, administration of MSCs, EPO or MSCs+EPO could rescue lung damage in hypoxia-induced BPD neonatal mice, more pronounced protection was found in mice treated with MSCs+EPO. This mechanism may be associated with inhibition of TGF-β1 signaling pathway to further suppress EMT process. Thus, MSCs, EPO, particularly MSCs+EPO, might be a promising therapeutic target for the treatment of BPD.

## RESULTS

### Characterization of cultured MSCs

**Figure 1:**
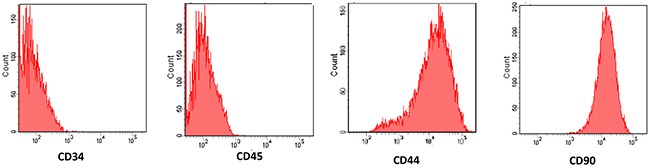
Characterization of cultured mesenchymal stem cells (MSCs) The surface markers were determined by fluorescence activated cell sorting (FACS) showed that MSCs showed positive expression of the surface markers CD44 and CD90, but negative expression of the hematopoietic markers CD34 and CD45.

After being primarily cultured, the MSCs appeared as spindle-like cells and attached to the tissue culture dishes. Three days after being subcultured, the cells were attached to the culture dish tightly and proliferated rapidly in the culture medium. The surface markers were determined by fluorescence activated cell sorting (FACS) showed that MSCs showed positive expression of the surface markers CD44 and CD90, negative expression of the hematopoietic markers CD45 and CD34. Figure 1).

### Changes of body weight, alveolar structure and and fibrosis

**Figure 2:**
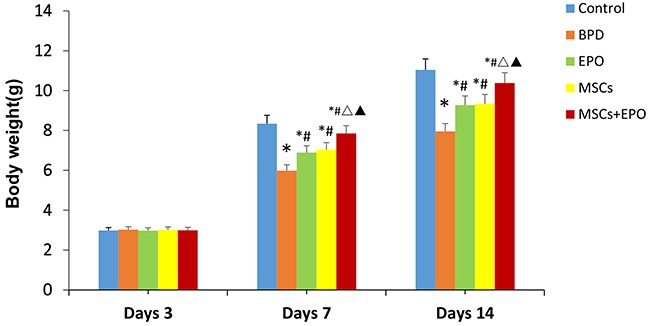
Comparison of body weight at different time periods in each group The data are present as mean ± SD (n=10). ^*^P<0.05 compared with control group;^#^*P*<0.05 compared with BPD group;^Δ^*P*<0.05. ^Δ^*P*<0.05 compared with EPO group. ^▲^*P*<0.05 compared with MSCs group. BPD: bronchopulmonary dysplasia, MSCs: mesenchymal stem cells, EPO: erythropoietin.

Neonatal C57BL/6 mice that had received injections of MSCs, EPO or MSCs+EPO at 1h before and 7d after hyperoxia-exposed, the same amount of phosphate-buffered saline (PBS) injection instead in BPD group. At 3,7 and 14 days after administration, the average body weights was lower in the BPD group than in control group, but was higher in MSCs, EPO or MSCs+EPO groups than in BPD group, respectively. Obviously, the average body weights more higher was shown in MSCs+EPO co-treatment mice than in MSCs or EPO alone (*P*<0.05, Figure 2).

**Figure 3:**
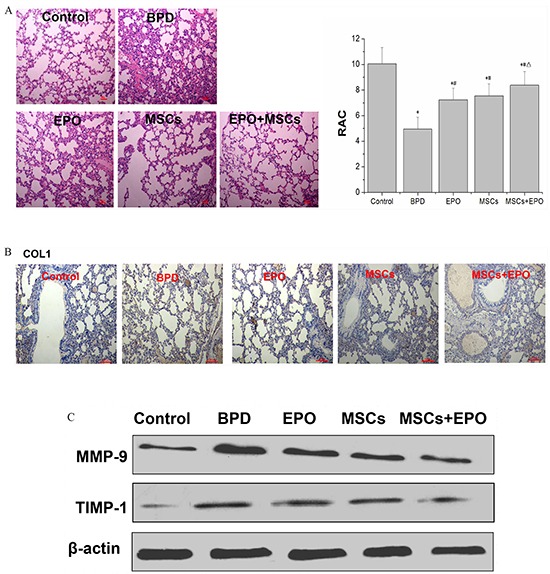
Hyperoxia impairs lung structure and fibrosis in neonatal mice **A.** Effects of MSCs, EPO or MSCs+EPO treatment during hyperoxia on radial alveolar counts (RAC). **B.** Immunohistochemical staining analysis of collagen type I (COL1) (magnification×100). **C.** Western blot analysis the protein expression of metalloproteinase-9 (MMP-9) and metalloproteinases-1 (TIMP-1) in lung tissue. The data are present as mean ± SD (n=10). ^*^P<0.05 compared with control group;^#^*P*<0.05 compared with BPD group. ^Δ^*P*<0.05 compared with EPO group.^▲^*P*<0.05 compared with MSCs group. BPD: bronchopulmonary dysplasia, MSCs: mesenchymal stem cells, EPO: erythropoietin.

Radial alveolar counts (RAC) is an important means of evaluation of alveolar development degree. In the neonatal mice exposure to hyperoxia at 14-days, lung histologic results showed that RAC was lower in BPD group (positive control) than normal group (control), but higher in MSCs, EPO and MSCs+EPO (treatment groups) in comparison with BPD group. Moreover, a better preservation of RAC was shown in MSCs+EPO co-treatment group than MSCs or EPO group alone (*P*<0.05, Figure 3A).

**Figure 4:**
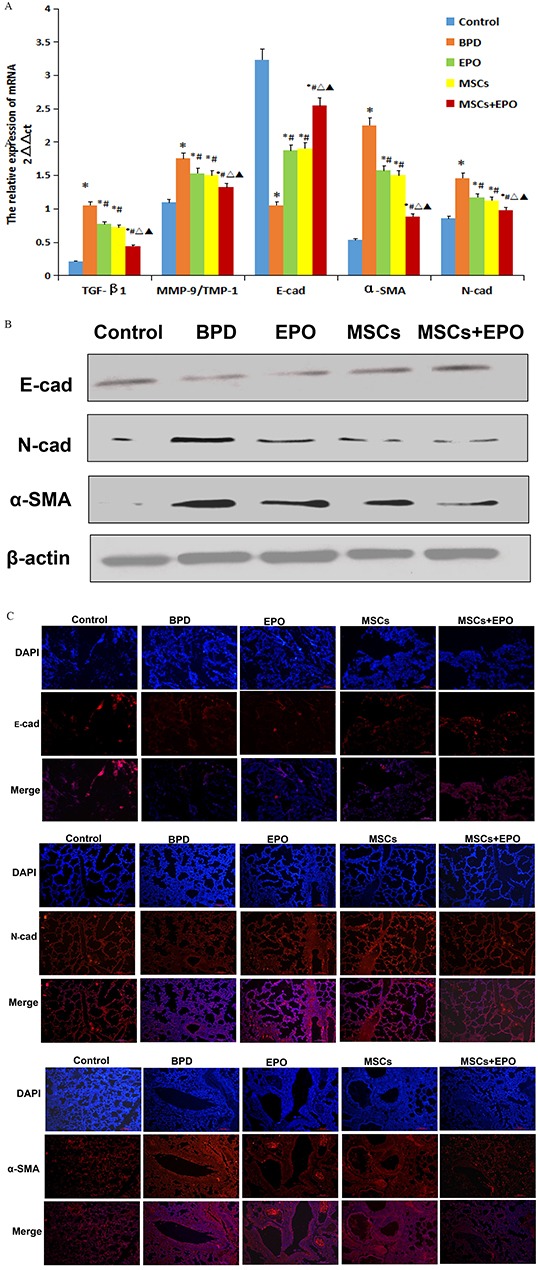
Effect of MSCs, EPO and MSCs+EPO on EMT markers in lung tissue **A.** Quantitative real-time PCR analysis of mRNA levels, **B.** Western blot analysis the protein expression. **C.** Immunofluorescence staining (magnification ×100). Merged image of DAPI with E-cad, N-cad and α-SMA staining respectively showed pink fluorescence. The data are present as mean ± SD (n=10). ^*^P<0.05 compared with control group;^#^*P*<0.05 compared with BPD group. ^Δ^*P*<0.05 compared with EPO group. ^▲^*P*<0.05 compared with MSCs group. TGF-β1: transforming growth factor-β1, E-cad: E-cadherin, N-cad.: N-cadherin, α-SMA: α-smooth muscle actin. TIMP-1: metallo-proteinases-1, MMP-9: metalloproteinases 9. BPD: bronchopulmonary dysplasia, MSCs: mesenchymal stem cells, EPO: erythropoietin.

The fibrosis of lung was detected by assessment the level of collagen type I (COL1), metalloproteinase-9 (MMP-9) and metalloproteinases-1 (TIMP-1). Immunohistochemical staining as shown in Figure 3B, western blotting and qRT-PCR as shown in Figure 3C and Figure 4A. The results indicated that positive expression of COL1, mRNA and protein expression of MMP-9/TIMP-1 in lung tissue was increased in the neonatal mice exposure to hyperoxia induced BPD group than in control, but reduced in MSCs, EPO, particularly MSCs+EPO group in comparison with BPD mice (*P*<0.05). Taken together, MSCs, EPO alone, especially MSCs+EPO were more effective in repairing hyperoxia-induced alveoli dysplasia injury.

### Detection of EMT markers

qRT-PCR, western blotting and immunofluorescence techniques were used to assess EMT-related proteins (E-cadherin, N-cadherin and α-SMA). As shown in Figure 4A, 4B and 4C, the protein expression level was in accordance with mRNA expression level. E-cadherin was significantly up-regulated, but N-cadherin and α-SMA were significantly down-regulation in three treatment groups when compared with BPD group (*P*<0.05). Moreover, the greater regulation was also shown in MSCs+EPO co-treatment mice than MSCs or EPO group alone. Epithelial cells express high levels of E-cadherin whereas mesenchymal cells express those of N-cadherin and α-SMA, decreased expression of E-cadherin and increased expression of N-cadherin and α-SMA is associated with EMT. These results revealed that MSCs, EPO and MSCs+EPO co-treatment were effective in inhibiting EMT process, importantly, MSCs+EPO had more inhibitory effects than MSCs or EPO alone.

### Detection of TGF-β1 signal pathway

**Figure 5:**
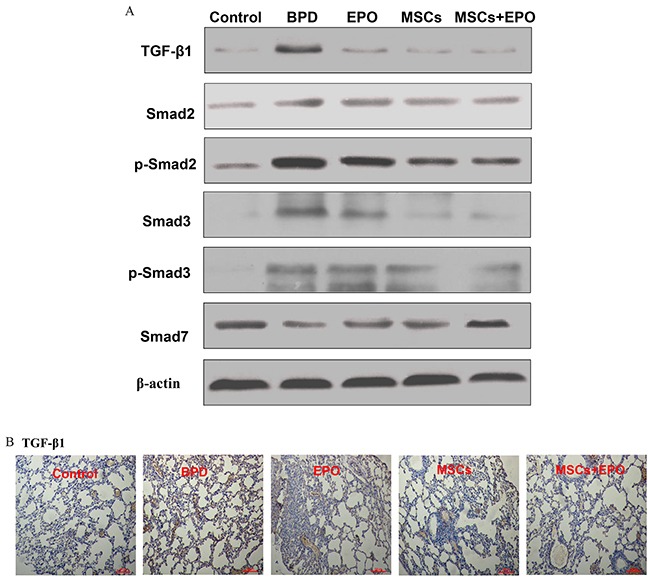
Effect of MSCs, EPO and MSCs+EPO on TGF-β1 signal pathway in lung tissue **A.** Western blot analysis the protein expression of TGF-β1, Smad2, p-Smad2, Smad3, p-Smad3 and Smad7. **B.** Immunohistochemical staining analysis of TGF-β1 (magnification ×100). BPD: bronchopulmonary dysplasia, MSCs: mesenchymal stem cells, EPO: erythropoietin.

To investigate the inhibition mechanism of MSCs, EPO and MSCs+EPO on the EMT, TGF-β1 signal pathway-related proteins (TGF-β1, Smad2, p-Smad2, Smad3 p-Smad3 and Smad7) were detected in lung histology in hyperoxia BPD animals. 14 days after injection, treatment groups showed a reduction in TGF-β1 and the transcription regulators (Smad2, p-Smad2, Smad3 and p-Smad3), however activated the protein expression of inhibitory Smad7 in comparison with the PBS- treated mice (Figure 5A and 5B, *P*<0.05). Based on these results, suggested that MSCs, EPO, in particular MSCs+EPO co-treatment could suppress TGF-β1 signaling pathway.

### Changes of blood vessels

**Figure 6:**
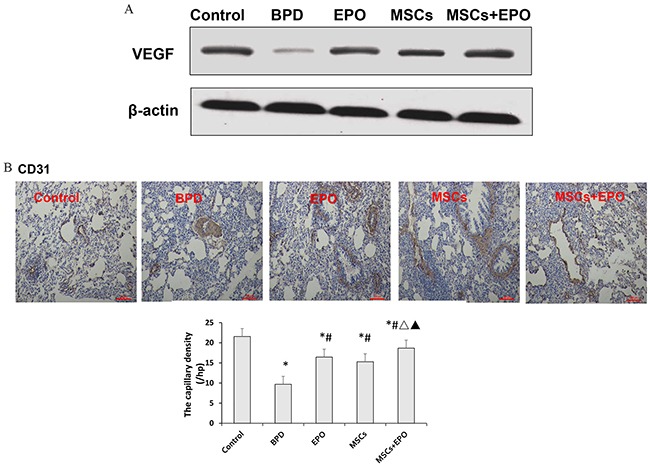
Effect of MSCs, EPO and MSCs+EPO on the blood vessels **A.** Western blot analysis the protein expression of vascular endothelial growth factor (VEGF). **B.** Immunohistochemical staining analysis of platelet endothelial cell adhesion molecule-1 (PECAM-1/CD31). The data are present as mean ± SD (n=10). ^*^P<0.05 compared with control group;^#^*P*<0.05 compared with BPD group. ^Δ^*P* <0.05 compared with EPO group. ^▲^*P*<0.05 compared with MSCs group. BPD: bronchopulmonary dysplasia, MSCs: mesenchymal stem cells, EPO: erythropoietin.

As mentioned before, the pulmonary capillary density was reduction in animal models and patients dying from BPD, enhancement of VEGF signaling could rescue the alveolar disruption induced by hyperoxia. To further explore the repair injury mechanisms, we analyzed the expression levels of VEGF and platelet endothelial cell adhesion molecule-1 (PECAM-1/CD31) by Western blotting and immunohistochemical staining. As shown in Figure 6A and 6B, proteins level of VEGF and vascular density were significantly increased in MSCs, EPO and MSCs+EPO treatment groups, individually. More importantly, a better increase in MSCs+EPO group than in MSCs or EPO treatment alone. (*P*<0.05).

## DISCUSSION

The present study for the first time demonstrated that MSCs, EPO alone especially MSCs combined with EPO may significantly promote lung repair after in hyperoxia-induced alveoli dysplasia injury, inhibition of TGF-β1 signaling pathway and suppression of EMT process.

MSCs are multipotent progenitor cells that can differentiation into multiple cell lineages [11]. An increasing amount of evidence has demonstrated that MSCs transplantation has become a potential therapy in many organs [12–16], such as MSCs therapy can effectively accelerate tendon-bone healing, alleviate the symptoms of neuropathic pain and resulted in subsequent motor recovery after spinal cord injuries, attenuate asthma by being phagocyted by lung macrophages, improve ventricular function in myocardial infarction and general well-being in patients with decompensated liver cirrhosis. Recently, there has been great interest in the potential therapeutic effect of stem cell as novel approaches to hyperoxic lung injury in neonatal rat [17, 18], and also MSCs can prevent arrested alveolar and vascular growth in part through paracrine activity [19, 20]. These may offer new therapeutic avenues for lung diseases, however, its potential role in the setting of neonatal lung injury have not been identified [21]. Erythropoietin (EPO), a glycoprotein hormone, a group of pharmacological agents with multifunctional effects. EPO was originally acknowledged as the main regulator of erythropoiesis, has anti-apoptotic, anti-inflammatory and anti-oxidative properties, the mechanism of organ-protective effects are not completely understood [22]. Moderate hyperoxia could decrease vessel density, impair lung structure, and reduce endothelial progenitor cells in the circulation and lung [23]. EPO has been suggested as a therapy for BPD, which can reduce the incidence of BPD in preterm infants, particularly treatment within the first 4 weeks of life [24]. BPD suffer from lung fibrosis secondary to myofibroblast-mediated excessive extracellular matrix (ECM) deposition and destruction of lung architecture. Fibrosis is characterized by excessive deposition of ECM, the accumulation of collagen, largely COL1 rich ECM is the hallmark of fibrosis. In addition, the turnover of ECM is partially regulated by proteases such as metalloproteinases (MMPs) and their inhibitors (TIMPs) [25]. In this study, we found that the expression of COL-1 and MMP-9/TIMP-1 were significantly decreased in lung tissue at 14-day injection of MSCs, EPO, especially, MSCs+EPO in BPD model, these results indicated that MSCs, EPO and MSCs in combination with EPO could reduce the lung fibrosis, interestingly, MSCs+EPO co-treatment more effective in repairing hyperoxia-induced alveoli dysplasia injury than MSCs or EPO treatment alone.

TGF-β1 signaling pathway play a crucial role during lung development [26]. Reports [27, 28] showed that the TGF-β is a positive regulator of myofibroblast in the alveolar septa and tracheal aspirate, TGF-β1 levels are increased in infants with BPD. Overexpression of TGF-β1 lead to structural changes, including proliferation α-actin-positive myofibroblasts within the alveolar septal walls and abnormal alveolar structure [29, 30]. On the other hand, TGF-β1 can induce cultured human type II alveolar epithelial cells (AEC2) through a epithelial-mesenchymal transition (EMT) into fibroblasts [31]. Mounting evidence indicates that alveolar EMT is primarily mediated by local production and activation of TGF-β1 [32–34]. In the present study, we investigated whether the effects of MSCs, EPO or MSCs+EPO on the repair hyperoxia-induced alveoli dysplasia injury mechanism was associated with TGF-β1 pathway and EMT process. The results showed that treatment groups downregulation mRNA and protein expression levels of epithelial marker E-cadherin and upregulation of mesenchymal markers N-cadherin and α-SMA. In addition, TIMP-1 induces expression of the developmental EMT transcription factors leading to downregulation of epithelial marker E-cadherin and upregulation of mesenchymal markers vimentin, N-cadherin, and fibronectin [35–37]. The study results showed the mRNA level of TIMP-1 was not consistent with the reports, one possible explanation may be associated with the disorders of MMPs/TIMP-1 in lung fibrosis. TGF-β1-activated Smad signaling induced EMT is a different way to fibrosis in BPD. The roles of TGF-β1-activated Smads in EMT have been confirmed in numerous studies, increased expression of Smad2 and Smad3 induces EMTs, while increased expression of Smad7 blocks TGF-β-induced EMT in multiple tissue[38–40]. TGF-β1 induced the receptor complex activation causes Smad2 and Smad3 activation through direct C-terminal phosphorylation. Phosphorylated Smad2 and Smad3 then form trimers with Smad4, and translocate into the nucleus, where they associate and cooperate with DNA binding transcription factors to activate or repress target gene transcription Consequently, Smad2 and Smad3 function in cooperation with Smad4 as TGF-β1 induced transcription regulators, on the contrary, the inhibitory Smad6 and Smad7 inhibit activation of the receptor-regulated Smads [41]. Further, the effect of MSCs, EPO and MSCs+EPO TGF-β1 signal pathway-related proteins were studies in lung histology in hyperoxia BPD C57BL/6 mice. Similarly, we found a reduction in TGF-β1 and the transcription regulators (Smad2, p-Smad2, Smad3 and p-Smad3), and a increase of the protein expression of inhibitory Smad7 in comparison with the PBS- treated mice. According to the results, we confirmed that MSCs, EPO, in particular MSCs+EPO co-treatment suppressed TGF-β1/Smads signaling pathway to further suppress EMT process. Though BPD is associated with extensive myofibroblast differentiation and extracellular matrix remodeling leading to lung fibrosis, the role of EMT as a major contributor to lung fibrosis is still heavily debated. There are reports that indicate that the lung pericytes rather than alveolar epithelial cells are the precursors of myofibroblasts during lung injury events. Although these, our experimental results demonstrated that MSCs, EPO in particular MSCs +EPO co-treatment attenuate EMT. These findings may explain the greater improvement and function in the mice treated with MSC+EPO than in those treated with MSC alone very early after injection. The exact mechanism of MSCs+EPO co-treatment attenuates EMT progression still incompletely known, such as whether have a time-dependent manner in the expressing switch from expressing epithelial cell to mesenchymal cell markers in the case of high oxygen induced bronchial injury, and whether have a correlation between MSCs+EPO combination with these changes. Those can not be clarified in the present study and would need further investigations.

According to reports [42–44], in the lungs of premature infants and animals who died with BPD, the expression of VEGF is decreased. Inhibiting VEGF receptor-2 (VEGFR2) causes rarefaction of pulmonary vessels and impairs alveolar formation in neonatal rats [45], whereas enhancement of VEGF signaling rescues the alveolar disruption induced by hyperoxia [46]. Thus, we detected the expression of VEGF and vascular density to further explore the mechanism of repair lung injury. In agreement with previous studies, proteins level of VEGF and vascular density were significantly decreased in BPD mice than control. These data were improvement in treatment groups individually, more importantly, a better increase in MSCs+EPO group than in MSCs or EPO treatment alone. The relationship between MSCs and EPO in the treatment of BPD is still not fully understood. EPO can stimulate the formation and differentiation of erythroid precursor cells in the bone marrow[47], enhance the beneficial influence of MSCs during recovery from tissue and organ injuries[48]. MSCs and endothelial progenitor cells (EPCs), express EPO receptor (EpoR) and/or mediate the proliferation of cells following EPO treatment [49, 50]. In addition, *in vivo* administration of EPO increases the number of MSCs in the bone marrow [51], transfected MSCs showed a significantly enhanced proliferation and migration [52]. Recently studies showed that blocking postnatal angiogenesis impairs alveolarization, and decreased pulmonary capillary density is observed in animal models and patients dying from BPD. How to promote blood vessels when MSCs in combination with EPO in hyperoxia-induced tracheal injury still remain to be clarified, a study on promote angiogenesis mechanism of MSCs+EPO co-treatment in BPD including migration and tubular structure formation ability of endothelial cells *in vivo* and vitro will be valuable.

In conclusion, the present study demonstrated that MSCs, EPO in particular MSCs +EPO co-treatment may improve hyperoxia-induced alveoli dysplasia injury at 14-day. This study for the first time suggest that EPO promoting protection of MSCs via inhibition of TGF-β1 signaling pathway related epithelial-mesenchymal transition process and the promoting angiogenesis effect. Even though this effect might be related to the addition of independent beneficial effects of the treatment agents, combination of MSC based therapy with pharmacy therapy might offer a novel therapeutic approach for the treatment of BPD.

## MATERIALS AND METHODS

### Animal and hyperoxia exposure

C57BL/6 mice were bought from the experimental animal center of the Fourth Military Medical University (Xian, China). 50 neonatal C57BL/6 mice (24h) were used in surgical procedure as the host animal. All animal procedures were approved by the animal ethics committee of Shandong University (Jinan, China) and followed the Guide for the Care and Use o f Laboratory Animals published by the U.S. National Institutes of Health (NIH Publication No. 85-23, revise d 1996). Experimental hyperoxia exposure was induced as previously described with some modifications [53], Newborn pups from different pregnant mice were allowed to deliver and recover in room air for 24 h. Mothers and pups were placed in Plexiglas chamber in which the oxygen concentration was maintained at a FiO_2_=0.21 (normoxia) or FiO_2_=0.60 (hyperoxia) for 14 days. Exposure to hyperoxia was continuous and cared for in a hypoxic environment, with a brief interruption for animal care (less than 10 min/day).

### MSC culture and transplantation

MSCs were isolated from the femur of 6-8-week-old C57BL/6 mice by using the method of whole bone marrow adherent cultures, and subsequently incubated with low glucose DMEM/F12 medium (Hyclone, USA) containing 10% fetal bovine serum (Becton, Dickinson and Company) at 37°C in 5% CO_2_. Fluorescence activated cell sorting was performed for analysis of MSCs immunophenotype as previously report [54]. Briefly, MSCs were suspended with trypsin and 5×10^5^ cells and were washed twice with PBS and then incubated with primary antibodies against rabbit CD34, CD44, CD45 and CD90 (Santa Cruz Biotechnology, Santa Cruz, California, USA). The second polyclonal antibody was added and incubated at 4°C for an additional 30 min in a dark room. A suspension of 1-5×10^5^ MSCs in 50 μl phosphate-buffered saline (PBS) was injected via intravenous administration or/and 5000U/kg recombinant human EPO by intraperitoneal injection respectively at 1 h before and 7 d after hyperoxia-exposed neonatal mice.

### Effect of MSCs/EPO on hyperoxia-induced alveoli dysplasia injury

#### Study design

24-hour-old mice pups were used in all experiments and were randomly devided into 5 groups (n=10 in each group): control, BPD, EPO, MSCs and MSCs+EPO group. The animals were euthanized with intraperitoneal injections of pentobarbital (100 mg/kg) at 14-day-old, the lung were quickly harvested and the left upper lobe was used in hematoxylin-eosin (H&E) staining and immunohistochemical staining, the left lower lobe was used in fluorescence staining, and the right lung was used for RNA and protein extraction.

#### Histological and immunohistochemical

Two weeks after operation, animals were anaesthetized as described previously and the left upper lobe were removed. Tissue samples were collected and fixed with 4% paraformaldehyde solution overnight, and then were embedded in paraffin. After deparaffinized in xylene and rehydrated by serial ethanol immersions in 100%, 95%, 85%, 75% and 100% water, the sections were cut with a microtome set at 4-5 μm (Leica RM226, Leica Microsystems, Heidelberg Germany). The cross-sections were stained with H&E (Baso Biotechnology, Shenzhen, China) and analysis of each section was carried out in a blinded fashion. Radial alveolar counts (RAC) is an important means of evaluation of alveolar development degree. Alveolarization was assessed by performing RAC, according to the method of Emery and Mithal [55, 56]. Briefly, from the center of the respiratory bronchiole a perpendicular was drawn to the edge of the acinus (as defined by a connective tissue septum or the pleura), and the number of septa intersected by this line was counted. Five counts were performed for each animal, average of the 5 high-power fields (hpf) was randomly selected. These studies were performed by two examiners blinded to treatment assignment.

To evaluate the effect of MSCs+EPO on lung fibrosis, angiogenesis and TGF-β1/Smad signaling pathway, the sections of each group were stained with rabbit anti-mouse polyclonal collagen type I (COL1; ab34710; Abcam, Cambridge, UK), transforming growth factor-β1 (TGF-β1; ab25121), CD31 (ab28364) or a nonspecific IgG antibody. For immunohistochemistry, 4-5 μm-thick cryosections were first blocked with 5% normal goat serum in PBS (ab7481; Abcam, Cambridge, UK) for 30 min. The sections were then incubated with the above mentioned primary antibodies overnight at 4°C and followed by incubation with 2-step plus®Poly-HRP anti-rabbit secondary antibody (PV-9000; ZSGB-Bio Co., Beijing, China) or in the dark with fluorescein isothiocyanate-conjugated goat anti-rabbit secondary antibody (ZF-0311; ZSGB-Bio Co., Beijing, China). Images were taken with a Nikon Eclipse 90i microscope. 10 serial sections (at an interval of 50 μm for each section) from each animal were used to quantify each parameter. The staining was analyzed with the image-analyzing system, Image Pro Plus 6 (Media Cybernetics, Rockville, MD, USA).

The degree of angiogenesis was determined by the capillary density of in the lung tissue by using a light microscope. Briefly, after staining the sections with the monoclonal rabbit antibodies CD31, five non-overlapping “hotspot” areas where the number of capillary was at a maximum in transverse sections were captured at low magnification (×100), and then the number of brown yellow capillary was counted in each of the five hotspot areas. The density was counted in blind on 50 sections and these studies were performed by two examiners blinded to treatment assignment.

### Quantitative real-time PCR

The right lung were frozen in liquid nitrogen and stored at −80°C. Total RNA was extracted from frozen samples, qRT-PCR analysis was performed to detect the relative pulmonary expression levels of metallo-proteinases-1 (TIMP-1), E-cadherin (E-cad), N-cadherin (N-cad) and α-smooth muscle actin (α-SMA) mRNA expression. The RNA sample was dissolved in RNase-free water and quantified spectrophotometrically. Primers were designed using the Primer Express software package (Applied Biosystems, Foster City, CA, USA): TGF-β1: 5′-GGCGGTGCTCGCTTTGTAC-3′(forward primer) and 5′-TCCCGAATGTCTGA CGTATTGA-3′(reverse primer); α-SMA: 5′-CTGTCCCTCTATGCCTCTGG-3′ (forward primer) and 5′-AGGGCTGTGATCTCCTTCTG-3′(reverse primer); E-cad: 5′-ATGGGGAAGCGGTGGAGGAT--3′(forward primer) and 5′-GTAGGCG ATGGCAGCGTT GTAG-3′(reverse primer); N-cad: 5′-GACCCAGAAGA TGATGT AAG-3′(forward primer); and 5′-CTCAGCGTG GATAGGC-3′(reverse primer); TIMP-1: 5′-TCCCCA GAAATCAACGAGACCACCT-3′′(backward primer) and 5′-A GAGTACGCCAGGGAACCAAGAAGC-3′(reverse primer); MMP-9: 5′-GGTGTTC TGCCAGACCAAGG-3′(forward primer) and 5′-TGCAAGATTGTCATCTTT A-3′(reverse primer); β-actin: 5′-TCTACAA TGAGCT GCGTGTG-3′(forward primer) and 5′-GGTCAGGATC TTCATGAGGT-3′(reverse primer). Data were analyzed with the ABI Prism 7900 sequence detection system software (version 2.2).

### Western blot analysis

The frozen right lung tissue were lysed using protein extraction buffer and equal amounts of protein were denatured and separated by sodium dodecyl sulfate-polyacryl-amide gel electrophoresis (SDS-PAGE). Protein concentrations were assessed using the BCA Protein Assay kit (Santa Cruz Biotechnology). 10μg of total protein were electrophoresed on 4-20% gradient SDS-PAGE gels and transferred to a nitrocellulose membrane. The membrane was blocked for 16 h at 4°C in blocking buffer containing 5% skim milk powder in TBST [20 mM Tris HCl (pH 7.4). Blots were probed with specific primary antibodies TGF-β1(ab25121), Smad2(ab63576), Smad3 (ab40854), and p-Smad2(ab188334), p-Smad3(ab52903), VEGF (ab46154), Smad7 (sc-11392; Santa Cruz Biotechnology, Inc), α-SMA (ab5694), E-cadherin (sc-7870), N-cadherin (sc-7939), TIMP-1 (ab61224). The goat anti-rabbit IgG (Boshide Inc., Shanghai, China) were incubated at 37°C for 1 h as the secondary antibody. Immunoreactions were visualized using an chemiluminescence (ECL) Western blotting kit (Amersham Biosciences) following the manufacturer′s recommendations. (Thermo Fisher Scientific Co., Ltd., Shanghai, China).

### Statistical analysis

All data are expressed as mean α SD. Statistical analyses were performed with 1-way analysis of variance (ANOVA) with a Dunnett's post-hoc test when appropriate by using SPSS version 13.0 statistical software. *P* <0.05 was regarded as significant statistical difference.
